# Engaging in purposeful patient and public involvement with young people living with obesity: recommendations from the ARROWS residential weekend

**DOI:** 10.1186/s40900-025-00776-2

**Published:** 2025-08-29

**Authors:** Elysa Ioannou, Megan Garside, Kath Sharman, Lucie Nield, Karen Coulman, Claire Woodward, Lousia Ells, Catherine Homer

**Affiliations:** 1 School of Sport and Physical Activity, Sheffield Hallam University, Sheffield, UK; 2 School of Health and Social Care, Sheffield Hallam University, Sheffield, UK; 3 Advanced Wellbeing Research Centre, Sheffield Hallam University, Sheffield, UK; 4 Obesity Institute, Leeds Beckett University, Leeds, UK; 5 SHINE, Sheffield, UK; 6 School of Medicine and Population Health, University of Sheffield, Sheffield, UK; 7 Population Health Sciences, Bristol Medical School, University of Bristol, Bristol, UK

**Keywords:** Obesity, Children and young people, Patient and public involvement, Methods, Lived experience, Evaluation

## Abstract

**Background:**

The ENHANCE (Evaluating the NHs engLANd Complications of Excess Weight Services for Children and Young People) study is a national evaluation of the Tier 3 complications of excess weight (CEW) services for children and young people living with obesity. Living with obesity can be linked to wider complex physical and mental health challenges, so it is crucial to incorporate the voice of those with lived experience to inform the evaluation. The value of involving patients and members of the public in research is recognised, however there are limited resources around how to meaningfully involve children and young people living with obesity in research, as well as a lack of practical guidance around delivery and impact.

**Methods:**

This article details the novel residential approach (including classroom sessions and teambuilding exercises) implemented in the ENHANCE evaluation to better engage young people and their families in the research. The aim was to build relationships, trust and confidence between attendees, and to learn from their lived experiences to inform the development of the evaluation. After the residential, attendees completed evaluation forms and de-brief sessions.

**Results:**

Eight young people, seven parents, one carer and six researchers attended a residential weekend in October 2024. The weekend included one overnight stay and a range of research and team building activities. Based on feedback from all attendees, the residential had a positive impact on families and helped to improve the evaluation. Feedback from families developed recommendations for designing residentials as part of study.

**Conclusion:**

This paper reports the process of designing the residential weekend, important considerations for delivery and the impact on young people, parents and carers with lived experience and the researchers. Advice and recommendations are provided to support other researchers to develop strong, innovative and purposeful patient and public involvement in their research.

## Abstract (plain language summary)

ENHANCE (Evaluating the NHs engLANd Complications of Excess Weight Services for Children and Young People) is a national evaluation of Tier 3 weight management services for children and young people across England. Living with obesity can be linked to many physical and mental health challenges, and so it is important to include those with lived experience in this evaluation. The value of involving members of the public in research is well-known, however there is often a lack of practical guidance around how to effectively involve children and young people in research studies.

The ENHANCE study team organised a residential trip to bring together patient and public involvement members. The aim was to build relationships, trust and confidence between attendees, and to learn from their lived experiences.

Eight young people, seven parents, one carer and six researchers attended a residential weekend in October 2024. The weekend included one overnight stay and a range of research and team building activities. Based on feedback from attendees, families enjoyed attending and the discussions and activities over the weekend helped to improve the design of the research.

This paper explains how the residential trip was designed and planned. It also explores the impact on young people, parents and carers with lived experience, the researchers and the impact on the evaluation. Advice and recommendations are provided to support other researchers to use valuable patient and public involvement activities.

## Introduction

In England, 2.6% of 4–5-year-olds and 5.5% of 10–11-year-olds are living with severe obesity [1]. Experiencing obesity in childhood is associated with physical and mental health challenges [2]. Additionally, many young people have negative experiences of weight stigma [3]. Complications of Excess Weight (CEW) clinics have been commissioned by NHS England as part of their commitment to treat children and young people (CYP) living with severe obesity identified in the NHS Long Term Plan [4]. CEW clinics aim to deliver holistic multi-disciplinary support to CYP (aged 2–17 years) living with obesity and its associated complications [5].

As the CEW clinics are relatively new (established 2021), their impact is currently being evaluated through this ENHANCE (Evaluating the NHs engLANd Complications of Excess Weight Services for Children and Young People) study, which will help inform the evidence base, optimise care, and support future delivery and commissioning [6]. Patient and Public Involvement (PPI) refers to research that is done ‘by’ or ‘with’ members of the public rather than ‘for’, ‘to’ or ‘about’ them [7]. So, the engagement and involvement of CYP and their families in ENHANCE is critical. On a wider scale, the importance of including CYP in research is recognised, with an understanding of the benefits for young people themselves as well as for researchers and research delivery [8, 9]. The United Nations Convention on the Rights of the Child (UNCRC), specifically Article 12 highlights that it is a human right for children to be involved in decisions about things that affect them [10]. This has led to increasing awareness of, and involvement of young people in health research [11].

Appropriate involvement of CYP in research requires careful planning, resourcing (e.g., costs, staffing) and flexibility, and if not considered can lead to ineffective or tokenistic involvement [12]. Whilst there are existing guidelines and frameworks for public involvement in research [13–15] these are often less well-developed for CYP, particularly for more vulnerable CYP [16]. The value in sharing more examples of what can be expected in practice has also previously been acknowledged [17, 18]. Therefore, this paper will share practical experience, providing reflections on a novel method of PPI in research with CYP.

The process taken to plan, deliver and reflect upon a residential experience will be discussed, including the innovative methods taken to support effective PPI in research with vulnerable young people. This paper will also reflect on whether such innovative methods can have a positive impact on the evaluation process and whether they are beneficial for CYP, their families and/or carers involved in studies.

### Establishing a PPI group: the ARROWS

As part of the ENHANCE project, a PPI group was established, comprising of families from various backgrounds with the commonality of experiencing care and treatment of CEW services and/or living with severe and complex obesity and comorbidities. The recruitment of initial members of the PPI group was supported through collaborating with SHINE, a community-based Tier 3 programme for CYP living with severe obesity, where author KS led the PPI efforts. PPI members were also directly recruited through researcher outreach activity at CEW clinic sessions and speaking to families that attended about ENHANCE and what participation in the group would involve. This became a core group for sharing their lived experiences to inform and guide the study, evaluate current provision, and improve the services received.

**Fig. 1:**
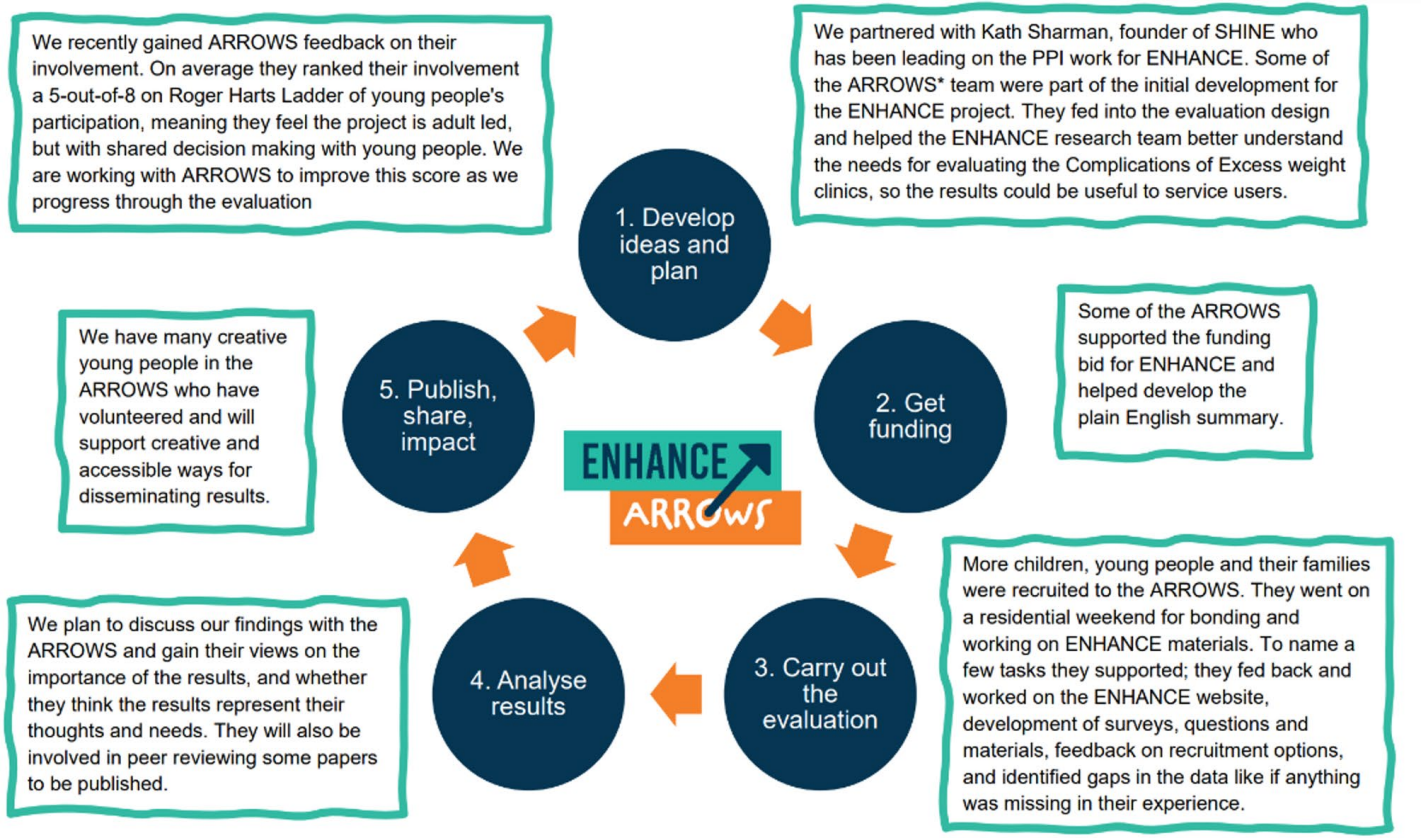
This diagram explains how young people can get involved in the ENHANCE project

The group was first established in 2022 and supported the bid writing stage (*N* = 7; CYP = 4, Parents = 3). The group grew with more members joining after the project was officially initiated in January 2024 and ‘branded’ themselves ARROWS, which stands for ‘Advocating for Responsible Research Opportunities for Wellbeing Services’. ARROWS includes 10 families who have accessed CEW clinics in either the north (*n* = 7) or the south (*n* = 3) of England. Those attending the residential included nine CYP (*n* = 5 female, *n* = 2 male and *n* = 2 non-binary; age range 13–18 years old), eight parents and one carer (Table 1). Any active, discharged or disengaged CYP that had been CEW patients could be onboarded into ARROWS, with off-boarding decided on an individual basis through discussions in the research team regarding their suitability to remain in ARROWS, including safeguarding considerations. To date, no CYP has been off-boarded or turned away. Throughout the ENHANCE project, and from the very start, a commitment was made to ensure active, inclusive and intentional engagement with the ARROWS (Fig. 1). Figure 1 shows how young people are involved in the ENHANCE project. It is adapted based on the diagram developed by ARC West public contributor Louise Ting and the Young People’s Advisory Group (YPAG) published on the NIHR website [16].

**Table 1:** Summary of key characteristics of the ARROWS

Region	Role	Gender, Age.
1	CYP	Female, 13 years old.
1	Parent	Mother of CYP.
1	Parent	Father of CYP.
2	CYP	Female, 16 years old.
2	Parent	Mother of CYP.
2	CYP	Male, 13 years old.
2	Parent	Mother of CYP.
2	Parent	Mother of CYP.
2	Parent	Mother of CYP.
2	CYP	Male, 15 years old.
1	CYP	Female, 13 years old.
1	Parent	Mother of CYP.
3	CYP	Female, 15 years old.
3	Parent	Mother of CYP
3	CYP	Female, 15 years old.
2	CYP	Non-binary, 18 years old.
1	CYP	Non-binary, 13 years old.
1	Carer	Female.

*CYP*,* Child or Young Person*. Regions numbered for anonymity. Regions 1 and 2 were from the North of England and region 3 was from the South of England

Due to the geographical spread of the PPI group, most of the meetings and interactions were conducted via Teams. Ensuring meetings are practical and accessible was a key theme that emerged from engagement work done to provide recommendations to CEW clinics [19]. Through these meetings, the group had already alluded to many negative experiences they had encountered, but recalls were sometimes filtered or superficial, and the provision of emotional support was limited due to the remoteness of the meetings. Working with the ARROWS for six months had developed trusting relationships. However, a residential was anticipated to bond the group further, aligning with the work by Rigby et al., for which another theme to emerge was the importance of face-to-face sessions for building trust within these groups of young people [19]. The budget for bringing the group together face-to-face was considered from the very beginning, due to acknowledging the geographic spread and the need to bring people together ‘in one room’ to develop the bonds between members. ARROWS were keen to participate in a residential with a request that the agenda should have an emphasis on some ‘fun time’ to balance more formal sessions.

### Planning the residential: the process

Residential trips in schools have been shown to be effective for helping students feel positive, improve their confidence, and support relationship building between both staff and peers, while also supporting team working and problem solving [20, 21]. Therefore, a residential weekend was proposed to help build trust, group cohesion and learning from the families’ lived experiences and how this can inform the development of the evaluation. Without previous knowledge of (a) residentials being used as part of the research process; or (b) residentials involving families rather than ‘school students’, the research team worked to co-develop a framework as to how the residential could work, in lieu of previously published guidelines. Whilst planning and preparing the residential, the researchers and ARROWS worked to ensure a safe learning environment for all, considering the differing needs of the group.

KS led a VARK (visual (V), aural (A), read/write (R), and kinesthetic (K)) learning styles assessment for researchers and ARROWS members to facilitate designing the residential activities, in addition to regular engagement with the group [22]. The VARK assessment identified that, while researchers in ENHANCE preferred read or write styles, most CYP and their families involved in the ARROWS group favoured kinaesthetic learning. These insights into learning styles enabled work with the group to take form in a way that suited the ARROWS preference, shifting to hands-on or visual tasks where possible, to optimise involvement and engagement with the group. This further supported the idea of organising an active residential weekend.

The ARROWS were involved in planning the residential, which the research team (EI, MG, LN, CH and KS) facilitated. Various opportunities for the in-person weekend were presented to the ARROWS at one of their regular online meetings. A PowerPoint presentation was used to offer and share pictures of each of the opportunities along with a selection of available dates, and travel time from their respective cities. Options included a residential weekend at an activity centre (with three locations across Yorkshire available), or a weekend trip to Nottingham, Birmingham, Leicester or Doncaster. These weekend trips included an activity day at a centre such as Sea Life Centre, Safari Park, Science Museum, Space Centre, or Sherwood Forest. The ARROWS then discussed the options as a group and provided feedback both in the meeting and separately via email. The residential weekend in Dearne Valley, South Yorkshire was selected by all of the families, based on the opportunity for team building activities, convenience of accommodation provided on site and proximity to a train station, meaning travel was more accessible even for those travelling from further away (e.g. from the South of England).

### Planning the residential: considerations

When the evaluation team started to plan ideas for the residential, several considerations were discussed. Firstly, ssignificant planning was done to ensure a trauma informed and inclusive environment, creating a safe environment where all individuals felt comfortable and able to participate [23]. The forming–storming–norming–performing model of group development was first proposed by Tuckman who described phases to enable groups grow, face up to challenges, plan work and find solutions [24]. Groups working cohesively can miss out ‘storming’ phases and successfully create an effective group function. For the residential itself, this translated to having a group agreement in place during the introductions. The research team further discussed ways of working sensitively and compassionately, noting previous PPI meetings discovering lived experiences, noting the unpredictable outcomes and potential blind aspects of emotional life traumas. This translated to including; hug monkeys, stress balls, time out safe spaces and 1:1 therapeutic support; games and light activities like wink murder, logic games, catch the snake, marshmallow tower; de-briefing during and after the residential with continued support on unresolved issues; and building emotional resilience to pre-empt and prepare for such circumstances. Alongside their input into the residential, ARROWS were sent a detailed information pack in advance, so they would be prepared for what to expect when attending the residential. This included a timetable for the weekend, details about the venue, a list of things to bring, the risk assessment and ‘top trumps’ information card about each member of the research team who would be in attendance along with their contact details.

Not all activities were suitable due to weight restrictions, confidence levels and ability. The final suitable and inclusive, yet varied activities included were archery, a night walk, orienteering and fire lighting, which were intermingled with research focused sessions. These activities help move brain function from emotions, memory and recall (function of the brain’s limbic system) to right brain function of creativity and imagination as the two cannot work at the same time [25]. Appropriate risk assessments were put in place, both through the residential facility and Sheffield Hallam University guidelines, both regarding the physical activities to be undertaken, as well as the emotional needs of the families (e.g. ensuring there was a known point of contact, breakout rooms, alternative activities available). Moreover, the staff at the centre were made aware of the needs of the group and the risk assessment, and detailed conversations were had in advance with all guardians to note any specific considerations that were required.

Other practical considerations firstly included organising branded ‘ARROWS’ hoodies for all the ARROWS CYP members and their parents to wear during the residential, and to take home, to support bonding and team cohesion. Secondly, the evaluation team had a discussion around food choices and options. While it was not possible to remove vending machines from the venue, MG liaised with the venue organisers about food options available to support inclusion of healthy food choices. Thirdly, some ARROWS members needed their siblings to attend, to permit attendance. This was accommodated as needed to ensure the group was cohesive while maintaining a balance around group size and costs of the weekend. Planning accommodation and rooms splits was not straightforward and depended on final numbers and availability. The end decision and availability permitted families to stay together in one room each, rather than splitting and mixing CYP and parents/carers. The inclusion of parents/carers to attend the residential was both because some were ARROWS members, but also because it enabled some ‘safety’ regarding safeguarding concerns as each CYP had a respective ‘guardian’ in attendance. The inclusion of consistent staff and a mental-health trained professional for the full duration of the residential further supported preparing for potential safeguarding concerns. Finally, there was a need to manage the inclusivity plan as one young person was not able to attend but wished to stay involved. As a result, online access was arranged to allow them to be involved in planning and group work sessions.

LN, CH, KS, KC, LE and MG attended the residential in person, either for the full weekend or one of the days, to support the activities and get to know the ARROWS better. CH is a female, White British, Academic and Public Health Specialist with experience of weight management service commissioning. LN is a female, white British academic and dietitian with experience of working in specialist weight management services. KS founder of SHINE has delivered community-based Tier 3 services for 22 years and is a Child and Adolescent Therapist. KC is a researcher and obesity specialist dietitian with experience of working in NHS weight management services. LE is a female professor of obesity, who also has a personal family history of obesity. MG is a female PhD student and a researcher with prior experience of working with young people and families and coordinating PPI activities through mental health research. All researchers had DBS checks, although this was not specific to this activity, as each CYP in attendance had a respective guardian. EI did not attend the residential in person but works with the ARROWS group regularly. She is researcher and a nutritionist with a background in sport science and has recently completed her PhD in diabetes and physical activity.

### The residential: timetable and research activities

The weekend began with ice breakers, using simple games in groups to facilitate developing relationships with each other. Throughout the weekend, desk-based work was interspaced with an opportunity for active or outdoor activities led by the site team. For example, fire lighting and orienteering were selected by the ARROWS. ARROWS were paid for their time contributing to ENHANCE specific activities only. Travel to the venue, the overnight stay, other activity costs and sustenance costs were covered by the research team through the project budget. The timetable of the weekend is presented in Table 2.

**Table 2:** Timetable of activities during the residential weekend

**Day 1**
**Travel to venue**
**Arrive** 12-1pm
**Welcome**,** lunch**, ** settle in** 1-2pm
**Introductions**,** Residential Brief and Icebreakers** 2-3pm (led by KS)
**Classroom Activity** *Clothesline Activity 3*-4pm
**Activity** *Archery* 4pm
**Dinner** 5:45pm
**Classroom Activity** *Feedback from Clothesline Activity* 6:45 − 7:45pm
**Activity** *Night Walk (Optional)* 7:45-10pm / Chat / Boardgames
**Day 2**
**Breakfast** 8:15am
**Classroom Activity** Group Tasks* (blog, website, lay summary, animation) 9:30 − 11:00am
**Activity** Bush craft (fire lighting and orienteering) 10:45am-12:45pm
**Lunch**,** debrief and certificates** 12:45pm
**Travel Home** 2pm

*Activities where ARROWS were paid for their time according to NIHR guidance

**Fig. 2:**
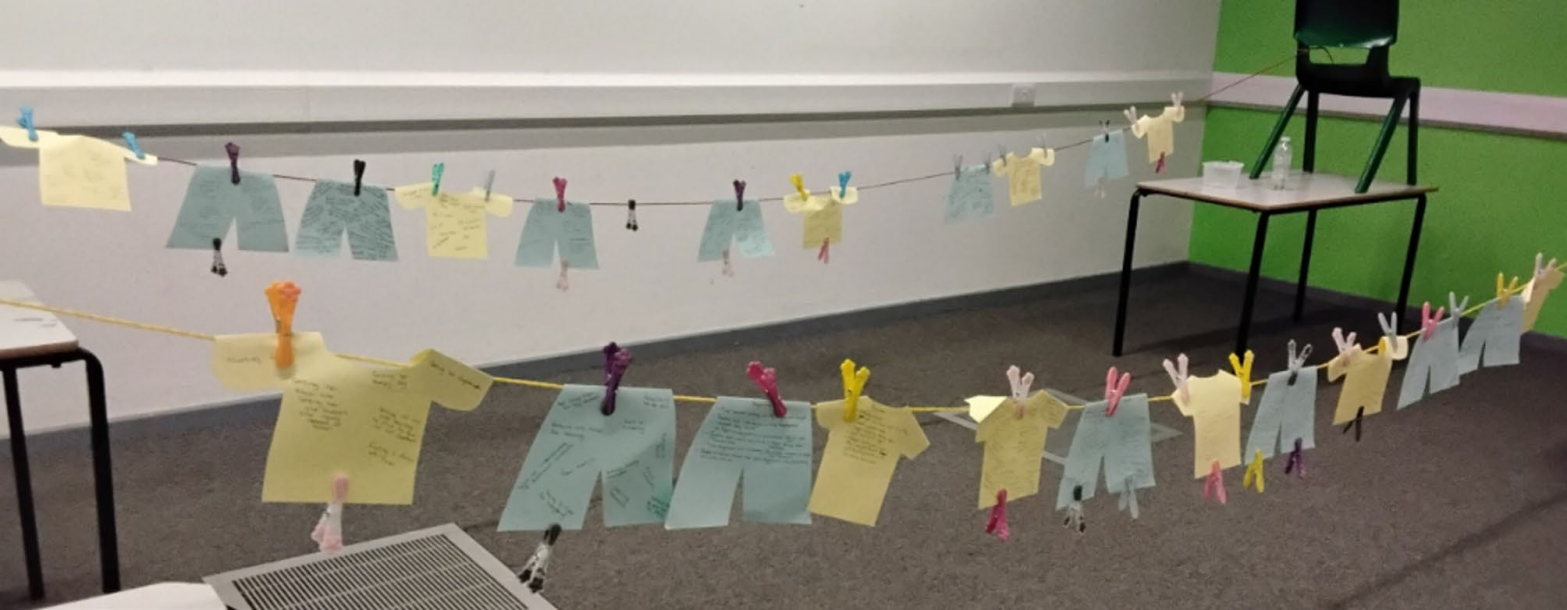
Image of the ‘shirts and pants’ activity [26]

In one activity there was an exploratory exercise around living with overweight or obesity where the group were asked to identify the positive experiences (written on a yellow shirt top cutout) and negative experiences (written on a green pants cutout) which had impacted on their life journey (Fig. 2) [26]. These comments were then ‘pegged’ onto a clothesline where it was clearly observable that negative experiences far outweighed the positives. In previous meetings with ARROWS, parents of CYP had mentioned in passing that they also needed support and a space to vent. It was also thought that the CYP would be more comfortable being open without their parents and other adults in the room. Therefore, for this activity only and specifically, it was decided to divide the group into separate spaces for adults and young people to recall experiences, without having to reflect on the consequences of their disclosures or filter their responses. This was followed by a de-briefing session and activity planning for the afternoon, another attempt at shifting brain function. Individual support was always available if and when needed.

On the second day, participants split into four small groups, each focusing on a different activity including designing the website, writing a lay summary of the project, creating blogs and reviewing a recruitment animation. This allowed for a variety of tasks (including more creative options as well as reading/writing), and allowed individuals the choice of what type of activity they were involved in.

The ARROWS members completed an evaluation form at the end of the weekend, which asked what they liked, disliked and what they would change about the residential (Table 3). This included Roger Hart’s Ladder of young people’s participation [27], to help understand the families’ perceptions on their involvement in ENHANCE and any areas for improvement (Table 4). After the residential, the researchers debriefed with the ARROWS through video and poster to avoid the potential risk of tokenism by acting on the views raised by young people, and continuing to engage and fully feedback to them in accessible ways about this [28]. ARROWS were further offered verbal 1-to-1 debrief sessions with KS, which they used to consolidate their experiences and found having some time after the residential to process emotional experiences before disclosing these as helpful for gaining closure. This reinforced the need for mental health professionals to be involved before, during and after such residentials. The researchers further debriefed, discussed and reflected on the weekend amongst themselves to gage the ‘impact’ the residential had on the ARROWS and on ENHANCE.

**Table 3:** The questions included in the evaluation form given to ARROWS at the end of the residential weekend

Number	Details
1	The things you/your child most enjoyed about the residential
2	The things you/your child enjoyed least about the residential
3	What would you/your child suggest to improve the residential
4	What did you/your child learn during the residential
5	Would you recommend the residential to other and why
6	Level of satisfaction of the residential (out of 10)
7	Any other comments

**Table 4:** The version of Roger Harts ladder of young people’s participation provided to ARROWS members

Level	Name	Description
1	Manipulation	Adults use young people to strengthen a cause and pretend that the cause is young person led.
2	Decoration	Young people are used to strengthen a cause, although adults do not pretend that the cause is young person led.
3	Tokenism	Young people appear to be given a voice, but in fact have little or no choice about what they do or how they participate.
4	Assigned, but informed	Young people are given a specific role and informed about how and why they are being involved.
5	Consulted and informed	Young people are consulted on adult-initiated projects. They are informed about how their input will be used and the outcomes of the decisions made by adults.
6	Adult led, decisions are shared with young people	Adults initiate projects but the decision-making is shared with young people.
7	Young person led and directed	Young people initiate and direct a project. Adult role is supportive as motivator/mentor.
8	Young person led, shared decisions with adults	Young people initiate projects and decision-making is shared between young people and adults. These projects empower young people while enabling them to access and learn from the experience and expertise of adults.

ARROWS were given this pictorially, on a ladder. The information has been extracted and inserted here

### Impact of the residential

Overall, the residential demonstrated similar impacts as those noted in the wider literature in terms of developing skills for young people and confidence, improving accessibility of the research and improving researchers understanding [29]. Changes in group dynamics evolved and altered over the two days, with increased engagement, trust and depth of relationships felt and observed, achieving the main aim of running the residential weekend. Specifically, one of the aims of the residential was to observe the personal developments of the ARROWS and improve the interpersonal relationships between the CYP, parents and carers in ARROWS, and the researchers. A discussion of what was observed and reflections on this, considering impacts on the young people in ARROWS, their parents or carers, the research and the researchers is considered in more detail below.

### Impacts on ARROWS members

Overall, the residential received positive feedback with ARROWS reporting that they enjoyed the activities and learning new skills, specifically enjoying the team-building activities and meeting the other ARROWS members face-to-face. Building up confidence and self-belief within the group during the residential appeared to help the ARROWS to take risks as the weekend progressed and become more invested in discussions and group work, disclosing deeper and sharing life experiences. ARROWS further reported gaining personal benefits from attending, including feeling less alone and being able to relate with each other. This aligns with the engagement work done recently for the CEW clinics, which highlights the importance of non-judgemental peer-to-peer support and improving mental health and wellbeing [19]. ARROWS fed-back that the residential helped them appreciate not to ‘judge a book by its cover’ and that they were not alone in their journeys, especially regarding those related to weight-loss services and other challenges faced by their peers. They all appreciated the opportunities to socialise, bond and grow:

*“We had such a lovely time it’s been a while since [name] felt normal and we didn’t stress about what she eats*,* does etc. We are very proud to be part of the study and as I mentioned I now have hope that these kids will not be stereotypically talked about. It was truly a wonderful experience I’ll treasure forever. Thanks again for the generosity*,* caring words and actions from all the team. ARROWS rock”* (Parent in ARROWS).

*“We really enjoyed the residential. Other families in the same situation helped us to realise we are not the only ones struggling and if the work we did can help other families get the help that they need*,* then that’s a bonus”* (Parent in ARROWS).

*“The residential was my first time of feeling hope. It truly was an amazing experience for us and we felt part of our new family. We are grateful and proud to be part of ARROWS”* (Parent in ARROWS).

*“I enjoyed the residential cos I got to make new friends and had fun”* (Young person in ARROWS).

*“The residential was brilliant to see and meet other families involved in person instead of on Teams. It was really good to hear and understand their stories and life’s challenges. It really was an eye opener. The activities were fun and we enjoyed the stay overnight.”* (Parent in ARROWS).

*“The residential was an exciting place / time to share my views and enjoy time and make friends.”* (Young person in ARROWS).

Aside from the benefits, ARROWS suggested how to improve future residentials such as, by debriefing straight after emotional sessions, and ensuring these are run after having more time to connect as a group. Despite having chosen the residential venue and type e.g. activity based, the ARROWS did suggest they would have liked more art activities. Other constructive feedback was related to factors outside of the ENHANCE team’s control, like wanting more comfortable beds and better weather. On average, the ARROWS ranked their satisfaction as eight-out-of-ten showing that they were very satisfied with the weekend. In terms of their role in ENHANCE, ARROWS rated their role as a six-out-of-eight on the Roger Harts Ladder of young people’s participation, meaning they feel ENHANCE is adult led, but with shared decision making with young people (Table 5).

**Table 5:** Summary of the ranked satisfaction of thoughts on participation style

	Level of satisfaction of the residential (out of 10)	Roger Harts Ladder of young people’s participation (from 1 manipulation to 8 CYP lead)
mean	8	6
median	8	6
mode	8	5
range	4.5 to 10	5 to 8

While the residential was aimed at building relationships and supporting PPI with CYP, including parents and providing the space to debrief seemed beneficial. The ‘pants’ (i.e., negative experiences) and ‘shirts’ (i.e., positive experiences) exercise was particularly helpful for parents, who completed this separately to their children. CYP’s ‘pants’ included weight stigma, bullying, overwhelming feelings, triggers, feeling scared and feeling overlooked, while their ‘shirts’ included ‘finding someone who cares’ in a healthcare context, getting the support they felt they needed and ‘feeling better’ since attending CEW.

Parents ‘pants’ reported more about how they had to deal with their own difficulties, mentally, physically, weight-based, medically, which impacted on their ability to support their child. They also seemed to have suffered through their own life of dealing with excess weight, stigma and difficulties, not being heard and feeling dismissed by healthcare professionals. Additionally, it was really difficult for them to see their children struggle, and they often reported they blamed themselves for this. Their ‘shirts’ were related to their children’s happiness, receiving support for their children or themselves and their own medical issues. They also linked to their own journey with weight cycling. Therefore, the residential was not only a positive and helpful experience for developing CYP interrelationships, but it was also a cathartic and helpful experience to their parents and carers. These social bonds and feelings felt by both parents and carers has undoubtably positively influenced the ENHANCE project which will be discussed further below.

### Impact of the residential on the evaluation process

Direct benefits to the ENHANCE study during the residential included the ARROWS support in the development of the ENHANCE study website [30], their filming of a voice over for a study animation and gaining feedback on recruitment and other study materials. This work was done over the course of the weekend, with quality output because of the bonding achieved, and the buzz of coming in from an activity – ‘the creative juices were flowing’. After the residential, the ARROWS felt more connected to each other. They have been more open, shared more during online sessions and have felt more involved and connected to the work, which has strengthened the feedback and support to the ENHANCE project due to the bonds and peer support they feel with each other.

An unexpected, yet important, outcome of the residential was the researchers understanding and bonding with the ARROWS which has led to more intentional and considered advocacy in the evaluation e.g. during delivery board meetings, outputs prepared, and considerations and decisions made. Attending the residential and being with the ARROWS for the weekend further developed the researchers understanding and appreciation of their experiences of obesity and CEW services. This deeper understanding and empathy from the research team because of attending the residential has been one of the most impactful responses to the residential on the ENHANCE project. This has allowed for more guided and improved discussions in study planning meetings, with these researchers more aware of and advocating on ARROWS behalf, considering the voice of CYP living with obesity as an integral part of ENHANCE, making the work more accessible.

### Impact of the residential on the research team

Taking part in the weekend was fun overall with the researchers enjoying the activities and hearing the positive feedback from families. However, hearing some of the difficult stories did impact the researchers, who also benefited from discussions and debriefs after the residential, to offload any concerns. The researchers also faced some anxieties over their responsibilities in safeguarding the CYP over the weekend and managing any potential conflict that could have occurred. Working over the weekend also placed burden on some researchers; by reducing the time they had for their own families. There was also some contention and challenges in ensuring there were enough staff and researchers available to work over the weekend, as it was important to keep consistent staff members for the benefit of the ARROWS, rather than covering the time in ‘shifts’. It was then challenging for the researchers to continue a normal work week after a tiring weekend, while also consolidating all the important information and learnings from the weekend and the feeling of completing a weekend that was time consuming and resource-demanding to plan first and foremost.

### Summary of learnings, reflections and recommendations

Although it is very difficult to pre-empt emotional reactions, and this unpredictability involves an element of risk taking, the residential has undoubtedly had a positive impact on the ARROWS and the research team. The research team worked hard to empower ARROWS, and were aware it was important to have an awareness of the existing power dynamics. However, the weekend allowed the ARROWS and the research team to get to know each other better and become a team of more equal influence. The depth of their disclosures could be attributed to how safe the group felt in their given environment and the strength of the trusting relationships that developed. Separating parents and young people during certain activities helped young people gain confidence and assertiveness, shifting towards more positive interactions. Learning from the residential supports the building of a strong awareness and foundation in evaluating present weight management services. Therefore, suggestions as to how CEW services can be improved to meet the needs of those living with weight related conditions can now be formulated based on real life experiences [10]. The residential benefitted the ARROWS and the ENHANCE project, and some recommendations based on learnings are described in Table 6 for future researchers to consider when planning engaging and purposeful PPI activities with CYP, filling an identified gap in reporting details of child involvement activities in health research [18].

**Table 6:** Recommendations for researchers for purposeful and effective PPI with CYP using a residential weekend

Recommendations ***for planning***	
1)	Budget generously for PPI activities and residentials for team building activities.
2)	Plan early and co-design the plan with attendees – involve them in decisions as much as possible.
3)	Ensure activities are risk assessed for any unique characteristics of the PPI group to ensure that there are multiple appropriate activity options available to choose from to help empower the CYP and their families.
4)	Ensure that specific room requirements can be accommodated and that there are single and multiple occupancy options available.
5)	Use the VARK tool to help understand needs of individuals, and tailor activities to them, including a mix of creative, active and written work.
6)	Consider whether individuals have the existing resource to take part in suggested activities (i.e. waterproof clothing) and provide/loan necessary equipment to CYP or families where possible.
7)	Create a comprehensive information pack, with timetable and packing list etc. to be prepared and sent in advance.
Recommendations ***for during***	
8)	Start the residential with fun, team-bonding activities so that people feel safe within the group, to enable people to share emotional or more challenging content - strengthen with a group agreement to provide a safe learning environment.
9)	Balance more emotive sessions with fun activities.
10)	Ensure consistent staff across the whole weekend.
11)	Ensure risk assessment and protocols are in place as above to ensure that during the weekend there is for example, a named point of contact for if anyone needs to debrief or take timeout. Ensure all staff (internal and external) are aware of needs of families and ensure staff are appropriately trained to support individuals and families with risk assessment and safeguarding during the residential and activities. At least one team member should be trained in mental health/counselling – especially with emotive topics and subjects planned. Have a plan for signposting onto other relevant services.
12)	Ensure flexibility in the timetabling of residentials to respond to energy levels, physical and emotional demands, debriefing and regrouping.
Recommendations ***for after***	
13)	The residential worked well in terms of having accommodation, activities and meals all in one place, but there may be some challenges with weather and accessibility of some activities. Evaluate residential activities and learn from experience, disseminate what worked well and what didn’t so other researchers can adapt their PPI approaches.
14)	Offer de-briefs with young people and their families.
15)	Make a plan and maintain connection with the group in a mode and frequency of communication that they chose.
16)	Researchers – remember to look after yourself and organise a debrief for researchers attending too, planning in for days off during the work week after the residential weekend.

## Conclusions

Residentials are a unique approach to enhancing the research process. They can help to strengthen the relationships between researchers and PPI members through being embedded in the weekend altogether, reducing barriers and promote understanding. Being conscious of inclusive and respectful communication methods can help improve interactions and lead to better collaborative relationships, which in turn addresses the power imbalance in research. Residentials can act as a creative method of collecting a deeper level of information if co-designed, well planned and compassionately managed. Giving young people a voice and valuing their contribution to research can support complex research questions, whilst also offering an opportunity for personal development of leadership and communication skills to be of value in later life.

## Data Availability

No datasets were generated or analysed during the current study.
